# Improvement in plasma D-dimer level in severe SARS-CoV-2 infection can be an indicator of fibrinolysis suppression: Case reports

**DOI:** 10.1097/MD.0000000000025255

**Published:** 2021-04-16

**Authors:** Daniel Manzoor, Chau Bui, Elias Makhoul, Daniel Luthringer, Alberto Marchevsky, Oksana Volod

**Affiliations:** Department of Pathology and Laboratory Medicine, Cedars-Sinai Medical Center, Los Angeles, CA.

**Keywords:** case report, coronavirus disease 2019, D-dimer, fibrinolysis shutdown, severe acute respiratory syndrome coronavirus 2, thromboembolism

## Abstract

**Rationale:**

Fibrinolysis shutdown associated with severe thrombotic complications is a recently recognized syndrome that was previously seldom investigated in patients with severe severe acute respiratory syndrome coronavirus 2 (SARS-CoV-2) infection. It presents a unique therapeutic dilemma, as anticoagulation with heparin alone is insufficient to address the imbalance in fibrinolysis. And while the use of fibrinolytic agents could limit the disease severity, it is often associated with bleeding complications. There is a need for biomarkers that will guide the timely stratification of patients into those who may benefit from both anticoagulant and fibrinolytic therapies.

**Patient concerns:**

All 3 patients presented with shortness of breath along with comorbidities predisposing them to severe SARS-CoV-2 infection. One patient (Patient 3) also suffered from bilateral deep venous thrombosis.

**Diagnoses:**

All 3 patients tested positive for SARS-CoV-2 RNA by reverse transcription polymerase chain reaction (RT-PCR) and were eventually diagnosed with respiratory failure necessitating intubation.

**Interventions:**

All 3 patients required mechanical ventilation support, 2 of which also required renal replacement therapy. All 3 patients were also placed on anticoagulation therapy.

**Outcomes:**

In Patients 1 and 2, the initial D-dimer levels of 0.97 μg/ml fibrinogen equivalent units (FEU) and 0.83 μg/ml FEU were only slightly elevated (normal <0.50 μg/ml FEU). They developed rising D-dimer levels to a peak of 13.21 μg/ml FEU and >20.0 μg/ml FEU, respectively, which dropped to 1.34 μg/ml FEU 8 days later in Patient 1 and to 2.94 μg/ml on hospital day 13 in Patient 2. In Patient 3, the D-dimer level on admission was found to be elevated to >20.00 μg/ml FEU together with imaging evidence of thrombosis. And although he received therapeutic heparin infusion, he still developed pulmonary embolism (PE) and his D-dimer level declined to 5.91 μg/ml FEU. Despite “improvement” in their D-dimer levels, all 3 patients succumbed to multi-system organ failure. On postmortem examination, numerous arterial and venous thromboses of varying ages, many consisting primarily of fibrin, were identified in the lungs of all patients.

**Lessons:**

High D-dimer levels, with subsequent downtrend correlating with clinical deterioration, seems to be an indicator of fibrinolysis suppression. These findings can help form a hypothesis, as larger cohorts are necessary to demonstrate their reproducibility.

## 1 Introduction

While the majority of patients with coronavirus disease 2019 (COVID-19) develop only mild respiratory illness and cough, approximately 14% require hospitalization and oxygen support and approximately 5% deteriorate into respiratory failure, requiring admission to the intensive care unit (ICU).^[1]^ In severe cases, these patients will develop acute respiratory distress syndrome (ARDS), sepsis, and multi-organ failure. Despite thromboprophylaxis, they also have a high incidence of thrombotic events (31%), most commonly thromboembolic (27%), with the majority being pulmonary embolism (PE).^[2]^ A constant finding in COVID-19-related ARDS is intra-alveolar fibrin deposition as well as macro- and microvascular thrombosis, with the latter being predominantly platelet/fibrin-rich.^[3,4]^ Fibrin deposition is the result of the dysregulation of the hemostatic balance between coagulation and fibrinolysis. The most common pattern of coagulopathy observed in patients hospitalized with COVID-19 is characterized by elevations in fibrinogen and D-dimer. Several studies from Wuhan have shown that markedly elevated D-dimer levels in COVID-19 patients are associated with higher mortality.^[1,5,6]^ Here we describe 3 fatal cases of severe acute respiratory syndrome coronavirus 2 (SARS-CoV-2) infection wherein patients developed severe venous thrombosis and/or PE despite improvement in their D-dimer levels.

## 2 Materials and methods

Retrospectively, we collected, reviewed, and categorized the clinical data of these 3 patients, including data on changes in coagulation parameters and postmortem findings. Possible mechanisms for the eventual downtrend of high D-dimer levels in SARS-CoV-2 patients with rapidly deteriorating clinical status are discussed. Informed consent could not be obtained, as all 3 patients had already expired by the time we began our investigation. A waiver for consent to publication was obtained from the Cedars-Sinai Office of Research Compliance and Quality Improvement (Institutional Review Board) Committee (approval number STUDY00001139).

## 3 Patient information

### 3.1 Patient 1

A 66-year-old obese female with history of cirrhosis (due to non-alcoholic fatty liver disease), chronic kidney disease, rheumatoid arthritis, and immune thrombocytopenic purpura, presented with 2 days of progressive shortness of breath and dry cough. She was found to be hypoxic with positive SARS-CoV-2 RNA by reverse transtcription polymerase chain reaction (RT-PCR). She was placed on high-flow nasal cannula but became increasingly hypoxic and hypercapneic, necessitating intubation on hospital day 4. She was treated with corticosteroids as well as ertapenem for possible comorbid bacterial infection. She was not a candidate for hydroxychloroquine therapy due to prolonged QT, and tocilizumab could not be given due to a platelet count of less than 100,000/μl. Despite treatment with prophylactic enoxaparin [initial D-dimer was 0.97 μg/ml fibrinogen equivalent units (FEU), normal <0.50 μg/ml FEU], she developed a rising D-dimer to a peak of 13.21 μg/ml FEU for which she was started on a continuous infusion of unfractionated heparin (UFH). Imaging studies did not reveal any thrombi in the lower extremities. Eight days later, her D-dimer level had dropped to 1.34 μg/ml FEU. She was persistently hypotensive requiring pressor support. She subsequently developed worsening anemia, thrombocytopenia, and multi-organ failure – lung, liver, and kidneys – requiring continuous renal replacement therapy (CRRT). She was transitioned to comfort care on hospital day 16 and expired on the same day.

### 3.2 Patient 2

A 58-year-old male with no significant past medical history presented with acute hypoxemic respiratory failure due to COVID-19. He was initially admitted to the medical ward but soon required ICU transfer for increasing oxygen requirements. He developed ARDS and was intubated on hospital day 8. His respiratory status remained tenuous throughout his admission, requiring the use of continuous paralytics, prone positioning, and pulmonary vasodilators. He received multiple investigative therapies for COVID-19 including hydroxychloroquine, tocilizumab, and Cardiosphere stem cells. Despite treatment with prophylactic enoxaparin (initial D-dimer 0.83 μg/ml FEU), on hospital day 6, he developed a rising D-dimer to a peak of >20.0 μg/ml FEU, similar to patient 1. He was found to have an acute, occlusive, superficial venous thrombosis in the right basilic vein. D-dimer level downtrended to 2.94 μg/ml on hospital day 13. Repeat ultrasound on hospital day 19 revealed that the thrombus had extended proximally, and that a new acute thrombus had formed in the right cephalic vein.

Due to thrombocytopenia and progressive thrombosis while on therapeutic UFH therapy, the patient was placed on argatroban and evaluated for heparin-induced thrombocytopenia (HIT). Heparin drip was resumed after a negative HIT screening assay [platelet factor 4 immunoglobulin G antibody, 0.08 optical density]. He was also found to have a right ventricular mural thrombus, which was treated with heparin infusion and a one-time dose of alteplase. He required broad-spectrum antibiotics and pressor support for persistent shock. He was also incidentally found to have latent tuberculosis, for which treatment was initiated. He was unable to be weaned from the ventilator, and after discussion with family, the decision was made to withdraw care on hospital day 37.

### 3.3 Patient 3

A 55-year-old male with history of type 2 diabetes mellitus, chronic kidney disease, diastolic heart failure, and hypertension, presented with lethargy and shortness of breath. He was intubated shortly after arrival on the date of admission and was confirmed to have COVID-19 by RT-PCR. He received tocilizumab as investigative therapy for COVID-19. He was also found to have *Klebsiella* pneumonia with a left loculated pleural effusion and positive culture, which was treated with antibiotics. On the date of admission, D-dimer was found to be elevated at >20.00 μg/ml FEU, and ultrasound revealed bilateral deep venous thromboses involving the peroneal, posterior tibial, and popliteal veins. UFH infusion was started at this time. Computed tomography angiogram of the chest on hospital day 25 later revealed nonocclusive pulmonary emboli involving the left upper, left lower, and right upper lobes of the lungs. By the time of this scan, D-dimer had declined to 5.91 μg/ml FEU. He developed worsening shock and lactic acidosis requiring vasopressor support and CRRT. The patient continued to decline despite maximal medical therapy, and the decision was ultimately made to withdraw care on hospital day 26.

Pertinent clinical, imaging, and laboratory findings for each patient are summarized in Tables 1 and 2.

**Table 1:** Clinical findings and treatment modalities.

	Patient 1	Patient 2	Patient 3
Demographic Characteristics			
Age	66	58	55
Sex	F	M	M
BMI/admission	36.3	22.9	25.9
Underlying medical conditions	Non-alcoholic steatohepatitis cirrhosis	Latent tuberculosis	Diabetes type 2; chronic kidney disease; hypertension; diastolic congestive heart failure
Previous thromboembolic disease	None	None	None
Days with COVID symptoms prior to admission	2 d	10 d	10 d
Hospital length of stay	16 d	37 d	26 d
Symptoms at admission	Shortness of breath, cough	Shortness of breath, subjective fever, dry cough, diarrhea, nausea, chest pain, loss of taste	Shortness of breath, lethargy
Overall length of disease	18 d	47 d	36 d
Imaging Features			
Chest X-ray	Bilateral hilar infiltrate	Interstitial infiltrating with lower lobe predominant	Bilateral infiltrate
Duplex ultrasound	LE, d 7: No DVT bilaterallyLE, d 15: No DVT bilaterally	UE, d 9: acute occlusive thrombus in right basilic vein LE, d 9: no DVT bilaterallyUE, d 20: progression of existing thrombus in right basilic vein; new acute occlusive thrombus in right cephalic vein LE, d 20: no DVT bilaterally	LE, d 2: Acute occlusive thrombus in the popliteal, posterior tibial, and peroneal veins bilaterallyLE, d 21: Subacute occlusive thrombus in the popliteal, posterior tibial, and peroneal veins bilaterally
Management			
Mechanical ventilation	Intubated d 4	Intubated d 8	Intubated d 0
ECMO	None	None	None
Renal replacement therapy	Yes	None	Yes
Treatment			
Antithrombotic on admission	UFH 5,000 U BID, switched to LMWH (prophylactic dose)	UFH 5,000 U BID, switched to LMWH (prophylactic dose)	UFH (therapeutic dose) + aspirin
COVID treatment	None	Hydroxychloroquine, tocilizumab, Cardisphere stem cells	Tocilizumab
Convalescent plasma	Yes	None	None
Corticosteroids	Hydrocortisone, methylprednisolone	None	None
Fibrinolytic agents	Alteplase 2 mg on day 16	Alteplase 50 mg on day 20	Alteplase 2 mg on day 5
Complications			
ARDS	Yes	Yes	Yes
Pulmonary embolism	Yes	Yes	Yes
Venous thrombosis	None	Yes	Yes
Acute kidney injury	Yes	None	Yes
Liver dysfunction	Yes	None	None
Other		Right ventricle mural thrombus	Septic shock

**Table 2:** Laboratory findings.

	Patient 1			Patient 2			Patient 3			
Laboratory Results	Admission	Mid/peak/nadir	Latest	Admission	Mid/peak/nadir	Latest	Admission	Mid/peak/nadir	Latest	Reference Intervals
SARS-CoV-2 RNA	Positive	Positive	Not done	Positive	Negative	Negative	Positive	Negative	Negative	Negative
LDH	541	618	2918	454	2428	558	824	324	Not done	125–220 U/L
IL-6	27.45	Not done	46.9	38.3	3702.2	123.5	26.1	Not done	Not done	<13.8 pg/ml
CRP	Not done	Not done	Not done	110.9	221.9	177.1	291.5	15.7	185.5	<5 mg/L
Ferritin	178	Not done	Not done	1255	1232.69	667.39	1722.83	1119.59	Not done	4.63–204.0 ng/ml
Procalcitonin	0.08	0.21	Not done	0.28	0.6	Not done	3.85	0.46	Not done	<0.07 ng/ml
Creatinine	1	1.49	0.91	0.8	1.12	0.72	9	1.2	3.23	0.57–1.11 mg/dl
AST	85	42	4202	84	144	32	44	44	21	5–34 U/L
ALT	37	29	1794	71	109	11	23	23	11	0–55 U/L
Hematology										
WBC	7.4	34.27	48.23	7.89	21.76	9.02	13.99	42.2	29.77	4.00–11.00 1000/U/L
Neutrophils	Not done	12.64	43.41	6.94	19.37	7.31	12.02	38.4	24.71	1.80–8.00 1000/U/L
Lymphocytes	0.17	1.03	0.96	0.61	0	0.36	0.99	0.26	1.45	1.00–4.50 1000/U/L
Platelets	77	159	39	161	107	324	338	317	212	150–450 1000/U/L
Coagulation Parameters										
PT (s)	14.5	18.1	39.2	14.5	14.1	13.7	19	Not done	Not done	11.9–14.4 s
aPTT (s)	32	Not done	43	31	50	44	31	Not done	Not done	22–37 s
Fibrinogen (mg/dL)	Not done	Not done	396	Not done	229	Not done	Not done	Not done	Not done	200–400 mg/dl
D-Dimer (FEU)	0.97	13.21	1.84	0.83	>20	3.05	>20	5.91	Not done	<0.50 ug/ml FEU

### 3.4 Summary of anatomic findings

Autopsies were performed on all 3 patients, where pathologic findings were mainly centered around the lungs. In each case, examination of the lungs showed diffuse alveolar damage (DAD, the histopathologic correlate of acute respiratory distress syndrome) in both the exudative (deposition of pink, fibrinous material lining the alveolar walls forming so-called “hyaline membranes”) and organizing (proliferation of type II pneumocytes and fibroblasts forming intraalveolar plugs and granulation tissue) phases, as well as squamous metaplasia of the smaller airways with mucus plugging. Necrosis was seen in the lungs of all patients, wherein 2 of whom it was associated with pneumonia and parenchymal infarction. Pulmonary vasculitis was present in 2 cases, one of which was associated with a lymphocytic response; lymphocytic infiltration was also seen in 1 case in an area of organizing diffuse alveolar damage. Numerous arterial and venous thromboses of varying ages, many consisting primarily of fibrin, were identified in the lungs of all patients, present in both large and small vessels. Based on the gross and histomorphologic findings, these are thought to be predominantly thromboembolic in nature, although areas are seen in all 3 cases that could certainly be consistent with in situ/intravascular thrombosis. Fungal forms morphologically consistent with *Candida* species were present in the lungs of 2 patients. Findings of clinical import in other organs thought to be related to the primary disease process were also seen. These included acute tubular necrosis with focal glomerular congestion and microthrombi in the kidneys along with focal ischemic necrosis of the liver in 1 patient (Patient 1), and myocarditis with interstitial edema as well as right ventricular dilation and mural thrombosis in the heart of another patient (Patient 2). Lymph nodes in 2 of the patients demonstrated plasmacytosis (Patients 2 and 3). Finally, the deep leg veins were dissected in 1 case to reveal bilateral deep venous thrombosis, confirming the clinical premortem impression (Patient 3). It should be noted that one of the cases was limited to examination of the heart and lungs only (Patient 2), and examination of the brain and spinal cord was not performed in any of the cases due to risks associated with the inevitable aerosolization of potentially infectious particles involved in removing the central nervous system. Pertinent autopsy findings for each patient are summarized in Table 3.

**Table 3:** Autopsy findings.

	Patient 1	Patient 2	Patient 3
Age/Sex	66F	58M	55M
Lung Pathology	DAD - exudative and organizing phasesBronchopneumoniaVasculitisNecrosisLymphocytic response in organizing DAD and vasculitisSquamous metaplasia with mucus plugs	DAD - exudative and organizing phasesNecrotizing pneumoniaInfarctsLymphocytic response in interstitial inflammationSquamous metaplasia with mucus plugs	DAD - exudative and organizing phasesNecrotizing pneumoniaLarge infarctFocal vasculitisSquamous metaplasia with mucus plugs
Thrombosis	Arterial and venousLarge and small vesselFibrin-predominant	Arterial and venousLarge and small vesselFibrin-predominant	ArterialLarge and small vesselFibrin-predominant
Infectious processes	Candida	None	Candida
Other organs	Kidneys: ATN, focal glomerular congestion with microthrombiLiver: Focal ischemic necrosis	Heart: myocarditis; mural thrombosis; RV dilation; interstitial edemaLymph nodes: Plasmacytosis	Lymph nodes: PlasmacytosisBLE DVTs
Notes	No brain	Heart/lung-only autopsy; no brain	No brain

## 4 Discussion

Here we described 3 patients who developed either new or worsening thrombosis despite prophylactic anticoagulation with low molecular weight heparin (LMWH) or therapeutic anticoagulation with UFH, and still succumbed to COVID-19-related complications. Notable in all 3 patients were initially high D-dimer levels that downtrended as their clinical conditions worsened as well as the development of extensive DAD. Numerous arterial and venous thromboses of varying ages, many consisting primarily of fibrin, were identified in the lungs of all patients on postmortem examination. They were present in both large and small vessels.

### 4.1 COVID-19 coagulopathy

**Figure 1:**
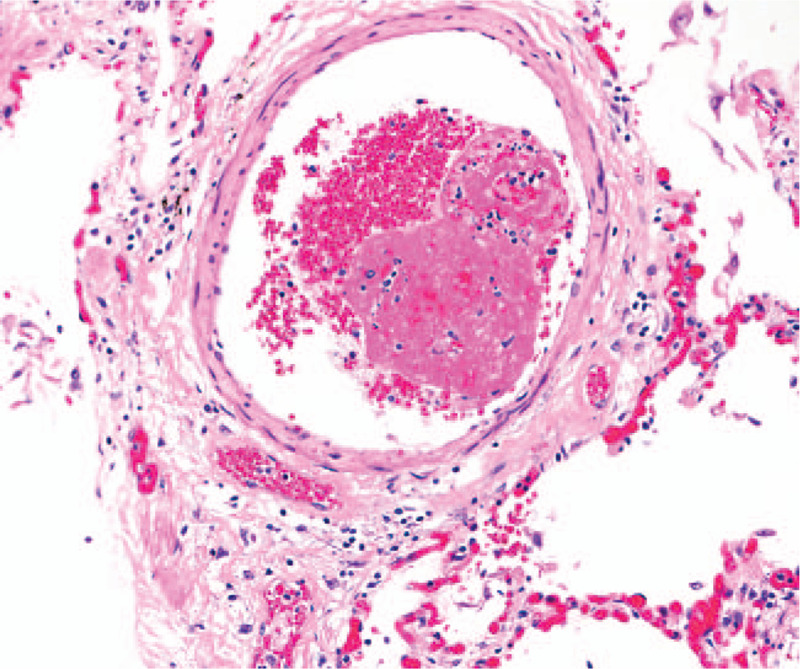
Histologic findings of lungs. Section of lung parenchyma demonstrates a small pulmonary venule containing a non-occlusive thrombus composed of fibrin and platelets with loosely attached red blood cells. There is no vasculitis (hematoxylin and eosin stain, 200x magnification).

COVID-19 coagulopathy is characterized by a prothrombotic state which appears to be different from the typically overt, sepsis-induced disseminated intravascular coagulation (DIC). Usually seen in non-COVID-19 sepsis-induced DIC, the early stage of DIC is characterized by hypercoagulability with secondary fibrinolysis, and the late stage of DIC is associated with hypocoagulability along with consumption of pro- and anticoagulant factors.^[7]^ Two distinct phenotypes of thrombotic complications can be seen in COVID patients: the usual “thromboembolic” type seen in other types of sepsis, and the diffuse micro-thrombotic type, predominantly seen in the lungs, but sometimes extending to other organs.^[8]^ The second type targets primarily the lungs and consists of widespread micro-thrombosis triggered by the cytokine “storm” that activates the coagulation cascade via a complex activation of the contact pathway (factor XII, factor XI, prekallikrein, high-molecular-weight kininogen [HMWK]), and not via the usual activation by way of tissue factor/factor VII.^[8,9]^ Factor XII is classified as a coagulation factor, but it is structurally related to tissue plasminogen activator (tPA), urokinase-type plasminogen activator (uPA), and plasminogen. While not as potent as its structural analogs, factor XII can also directly activate plasminogen.^[10]^ Kallikrein stimulates the generation of bradykinin, which induces the release of inflammatory cytokines leading to complement activation, promoting fibrin and cellular deposits in the microvasculature. This pattern has been defined as thrombo-inflammation and is a distinct form of DIC.^[11]^ The activation and dysfunction of pulmonary endothelial cells induced by SARS-CoV-2 contributes significantly to its pathogenesis. This is seen mostly in patients with comorbidities causing chronic endothelial dysfunction (e.g., diabetes, hypertension, and other cardiovascular disease). A useful review regarding the proposed cellular and molecular mechanisms of endothelial activation and dysfunction in the context of COVID-19 has been recently published.^[12]^ It outlines that hemostasis relies on a balanced interaction of endothelial cells, platelets, coagulation factors, anticoagulants, and other factors involved in fibrinolysis. The endothelium plays a key role in the regulation of this hemostatic balance. As a result of vascular injury, endothelial cells have the ability to confine clot formation to the site of the injury and are involved in all the major hemostatic pathways. Endothelial system disruption or damage may result in bleeding or clotting diatheses. In COVID-19 infection, cytokines released by endothelial cells in addition to SARS-CoV-2 itself can lead to endothelial activation and dysfunction, disruption of vascular integrity, and endothelial cell apoptosis, creating a thrombogenic platform for coagulation pathways as well as production and activation of platelets. Resultant edema, inflammation, and microthrombi are hallmarks of COVID-19-associated ARDS and can be demonstrated in the autopsy findings of all 3 of our examined patients (see Table 3, Fig. 1). These microthrombi may then escape into the general circulation and increase the risk of deep vein thrombosis, pulmonary embolism, and stroke.

Especially in those with severe disease, COVID-19 coagulopathy has been emerged as a key factor of high morbidity and mortality. As seen in our cases, the combination of both phenotypes can induce severe, potentially lethal disease.^[2,8,13]^

### 4.2 Fibrinolysis impairment in COVID-19

Unlike hyperfibrinolysis seen during the prothrombotic stage of sepsis-induced DIC, septic COVID-19 patients commonly display only minimal fibrinolysis when viscoelastic assays are utilized in their assessment.^[14,15]^ This “fibrinolysis shutdown” has been shown to correlate with thromboembolic events and the need for hemodialysis.^[15]^

The possible role of the fibrinolytic system in COVID-19 coagulopathy was recently published.^[16]^ In brief, SARS-CoV-2 utilizes angiotensin converting enzyme 2 (ACE2) as its receptor. ACE2 is present in the lungs, kidneys, endothelial cells, heart, gastrointestinal tract macrophages, and lymphocytes. Its abundance in type II alveolar cells and pulmonary endothelium renders the lungs as an important target organ for SARS-CoV-2. Fibrinolysis is impaired because of the dysregulation of the renin-angiotensin-aldosterone-system (RAAS).^[17]^ Angiotensin II induces the expression of plasminogen activator inhibitor-1 (PAI-1, suppressor of fibrinolysis) on endothelial cells.^[18]^ As ACE2 is downregulated following the attachment of SARS-CoV-2, this balance is shifted to an excess of angiotensin II, which, in turn, increases PAI-1. As described earlier, SARS-CoV-2 also triggers an acute inflammatory response with an increase in bradykinin which induces tPA expression (activator of fibrinolysis) in the endothelium.^[19]^ This increase in tPA is still not sufficient to counterbalance the extremely high levels of PAI-1 induced by the excess of angiotensin II. This results in inhibition of fibrinolysis and may also explain the fibrin deposits seen in the alveoli of the lungs.

Fibrinolysis suppression is also seen in sepsis induced DIC. It is usually associated with only slightly elevated D-dimer levels along with a marked increase in PAI-1.^[20]^ In fact, a decline in or normalization of the D-dimer level in septic patients has been hypothesized to indicate fibrinolysis inhibition and was associated with high mortality.^[21]^ A similar pattern was seen in our patients: their D-dimer levels “improved” while their clinical conditions worsened.

### 4.3 Laboratory assessment of fibrinolysis

D-dimer is a unique marker of coagulation cascade activation, fibrin clot polymerization, stabilization by factor XIII, and subsequent degradation by plasmin. Fibrin molecules that contain D-dimer are formed both extra- and intravascularly during hemostasis, thrombosis, and tissue repair processes. The clinical utility of D-dimer measurement has been most applicable in the exclusion of venous thromboembolism (VTE) and the diagnosis of DIC. The fact that only a small amount of circulating fibrinogen is needed to be converted into a fibrin clot and lysed by plasmin to generate a detectable D-dimer level makes this assay very sensitive. In the right clinical setting, a normal D-dimer level indicates that there is no major activation of intravascular coagulation. On the other hand, especially in hospitalized patients, the implications of the D-dimer level have low specificity as there are many other conditions or situations with ongoing activation of the hemostatic system in which D-dimer levels can be elevated. These include pregnancy, inflammation, malignancy, trauma, liver disease (decreased clearance), heart disease, sepsis, hemodialysis, or recent surgery.^[22,23]^ D-dimer testing alone is insufficient to make diagnostic decisions in VTE or DIC, therefore, it is more commonly used as part of a diagnostic algorithm. Markedly elevated D-dimer levels have been found to increase the likelihood of PE.^[24]^ Conversely, in septic patients, a decline in the D-dimer has been hypothesized to indicate fibrinolysis inhibition and is associated with higher mortality.^[21,25]^

There are 2 main types of D-dimer assays, each reporting different D-dimer units. The fibrinogen equivalent unit (FEU) reports D-dimer levels based on the molecular weight of fibrinogen, whereas the D-dimer unit (DDU) reports D-dimer levels based on its own molecular weight, which is about half that of fibrinogen. How the units are reported varies depending on the manufacturer, resulting in up to 9 different unit combinations: mg/L, mg/dl, ng/dl, ng/ml, μg/L, μg/ml, μg/dl, mg/ml, and ng/L. Although no specific cutoff is defined, a three-to-four fold increase in D-dimer levels should be considered as significant per the international society of thrombosis and hemostasis (ISTH) interim guidelines on the recognition and management of coagulopathy in COVID-19.^[26]^

In 2 of our patients (Patients 1 and 2), the initial D-dimer levels of 0.97 μg/ml FEU and 0.83 μg/ml FEU were only slightly elevated (normal <0.50 μg/ml FEU). They developed a rising D-dimer to a peak of 13.21 μg/ml FEU and >20.0 μg/ml FEU, respectively, which dropped to 1.34 μg/ml FEU (1340 ng/ml FEU) eight days later in Patient 1 and to 2.94 μg/ml (2940 ng/ml FEU) on hospital day 13 in Patient 2. In Patient 3, the D-dimer level on admission was found to be elevated to >20.00 μg/ml FEU together with ultrasonographic evidence of thrombosis. Despite therapeutic heparin infusion, he developed PEs and his D-dimer level declined to 5.91 μg/ml FEU (5910 ng/ml FEU). The markedly elevated D-dimer concentration seen in these patients was strongly suggestive of clot formation (Patients 2 and 3 had thrombosis documented by imaging) and subsequent plasmin-mediated fibrinolysis. On the other hand, as their conditions worsened and they developed deep vein thromboses (DVTs), PEs, and DAD (postmortem findings), it is reasonable to speculate that the D-dimer decline was an indicator of fibrinolysis suppression.

Fibrinolysis is a complex and dynamic process. There is no single conventional assay that considers the interactions between the proteins of the coagulation and fibrinolytic pathways, the cellular components of blood, and the vessel walls. Individual factors may be challenging to measure and assays are not readily available, even in major centers like ours, and often require several days to perform. We were able to send a blood sample from one of our patients (Patient 1), collected during the morning of her last day of life, to a reference laboratory for a limited fibrinolysis panel. As was postulated, we saw a significant increase in the PAI-1 level to >200 ng/ml (normal 4–43 ng/ml), increased tPA of 78.1 ng/ml (normal <12.8 ng/ml), decreased plasminogen activity at 38% (normal 65%–176%), and decreased alpha-2-plasmin inhibitor activity at 52% (normal 85%–156%). Although increased, the tPA level was likely still insufficient to counterbalance the extremely high PAI-1. Of note, it took more than 1 week to obtain these results. Not surprisingly, there is an interest in using viscoelastic hemostasis assays (VHAs) such as thrombelastography (TEG) or thrombelastometry (ROTEM), especially when there is a need for rapid assessment of fibrinolysis dysregulation.

### 4.4 Role of viscoelastic hemostasis assays (VHA) in fibrinolysis assessment

Unlike individual fibrinolytic assays, VHAs can provide a real-time, global evaluation of clot initiation, formation, and lysis, in a single graphic tracing. Moore et al. defined TEG-based “fibrinolysis shutdown” in trauma patients as a relative resistance to tPA due to dysregulation of the plasminogen-plasmin system. In their animal model experiments, they demonstrated that shock produced systemic hyperfibrinolysis, whereas tissue injury led to physiologic fibrinolysis shutdown. They stratified trauma patients into 3 different categories based on the TEG LY30 parameter: hyperfibrinolysis (≥3%), physiologic fibrinolysis (0.81%–2.9%), and fibrinolysis shutdown (≤0.8%).^[27]^ A recent study by the same group demonstrated “fibrinolysis shutdown” in critically ill COVID-19 patients. In their experience, a TEG maximal amplitude (MA) lysis of zero, that is, lysis occurring 30 minutes after MA is reached (MA LY30 of 0), together with a D-dimer of >2600 ng/ml FEU (normal <500 ng/ml FEU), was predictive of thromboembolic events and the need for hemodialysis.^[15]^ They propose this combination of findings as an indicator of fibrinolysis shutdown in COVID-19 patients. Whole blood clot lysis assays usually show evidence of lysis only when there is a high level of tPA that overcomes the inhibitory effect of PAI-1, as is usually seen in trauma or during cardiopulmonary bypass.^[28]^ In the authors’ published and unpublished experience, the treatment of patients with sepsis-related DIC fibrinolysis using UFH or LMWH resulted in normalization of the LY30 parameter and prevention of progression to DIC.^[29,30]^ Heparin decreases the rate of thrombin generation in the prothrombotic state of DIC and reduces inflammatory biomarkers.^[31,32]^ As all patients in the discussed TEG study were either on LMWH or unfractionated heparin, it cannot be concluded with certainty if an LY30 of 0, while on anticoagulation, indicates fibrinolysis shutdown or normalization of fibrinolysis. Other authors also questioned the conclusions drawn by Wright et al with respect to the association between high D-dimer levels and TEG in the setting of fibrinolysis shutdown in COVID-19 patients.^[33]^ Additional studies on COVID-19 patients who are not on anticoagulation are needed to confirm these conclusions, and the results should be correlated, at the very least, with corresponding levels in tPA and PAI-1.

## 5 Challenges to therapeutic approach

The coagulopathy associated with COVID-19 appears to be complex, as does its management.

It is evident that patients with COVID-19 do develop fibrinolytic abnormalities.^[8,15,34]^ They also have a high rate of VTE despite receiving therapeutic anticoagulation.^[2]^ Anticoagulant therapy with UFH or LMWH is essential to limit ongoing fibrin clot formation, but it is ineffective in clearing already formed fibrin deposits, which is normally done by the patient's own fibrinolytic system. Therefore, it is imperative to identify biomarkers of fibrinolysis shutdown to inform and allow clinicians, as soon as it is indicated, to use other modalities to reverse this process and improve patient outcomes.^[8,9]^ All 3 of our patients did receive a single dose of alteplase, but the dose was given late in the course of their disease and was not significantly effective.

## 6 Conclusion

Individual factors of fibrinolysis may be challenging to measure and are not readily available. VHAs are gaining more interest among clinicians and are available in several centers but are still used infrequently in COVID-19 patients, our institution included (TEG was not requested for any of our 3 patients despite being available). The significance of the TEG LY30 parameter in anticoagulated patients is unclear. D-dimer, however, is a readily available assay in all hospitals. For hospitalized patients, there is no consensus on how often D-dimer should be measured, or how the results should be acted upon with respect to anticoagulation in COVID-19 patients. From the presented cases, it appears that serial D-dimer measurements could be beneficial. In conclusion, it is not a distinct threshold level of D-dimer, but rather a high D-dimer level with subsequent downtrend correlating with clinical deterioration, that seems to be an indicator of fibrinolysis suppression.

## 7 Limitations

As mentioned earlier, individual factor assays are often not readily available, and turnaround times are substantial; our study certainly would have benefited from being able to measure these parameters on more than just 1 patient. And although TEG is readily available at our institution, it was not utilized by the clinical team in these cases. Lastly, because of the limited size of our cohort, our findings should be interpreted as a hypothesis that warrants further investigation. Of course, more data and greater numbers of patients are necessary to demonstrate the reproducibility of these findings.

## Author contributions

OV contributed to concept and design; AM, EM, DM, and DL performed autopsies and provided review and interpretation of postmortem findings; DM, CB, and OV extracted and analyzed pre- and postmortem data, prepared tables, reviewed the literature, and wrote the initial draft; DM and OV were responsible for manuscript revision. All authors issued approval for the final version to be submitted.

**Conceptualization:** Oksana Volod.

**Data curation:** Daniel Manzoor, Chau Bui, Elias Makhoul, Oksana Volod.

**Formal analysis:** Daniel Manzoor, Daniel Luthringer, Alberto Marchevsky.

**Writing – original draft:** Daniel Manzoor, Chau Bui, Oksana Volod.

**Writing – review & editing:** Daniel Manzoor, Daniel Luthringer, Alberto Marchevsky, Oksana Volod.
